# Recent Advances in the Pathophysiology of Fatty Acid Oxidation Defects: Secondary Alterations of Bioenergetics and Mitochondrial Calcium Homeostasis Caused by the Accumulating Fatty Acids

**DOI:** 10.3389/fgene.2020.598976

**Published:** 2020-11-27

**Authors:** Alexandre Umpierrez Amaral, Moacir Wajner

**Affiliations:** ^1^Programa de Pós-Graduação em Ciências Biológicas: Bioquímica, Instituto de Ciências Básicas da Saúde, Universidade Federal do Rio Grande do Sul, Porto Alegre, Brazil; ^2^Departamento de Ciências Biológicas, Universidade Regional Integrada do Alto Uruguai e das Missões, Erechim, Brazil; ^3^Departamento de Bioquímica, Instituto de Ciências Básicas da Saúde, Universidade Federal do Rio Grande do Sul, Porto Alegre, Brazil; ^4^Serviço de Genética Médica, Hospital de Clínicas de Porto Alegre, Porto Alegre, Brazil

**Keywords:** fatty acid oxidation defects, bioenergetics, calcium homeostasis, mitochondrial permeability transition, mitochondrial functions

## Abstract

Deficiencies of medium-chain acyl-CoA dehydrogenase, mitochondrial trifunctional protein, isolated long-chain 3-hydroxyacyl-CoA dehydrogenase, and very long-chain acyl-CoA dehydrogenase activities are considered the most frequent fatty acid oxidation defects (FAOD). They are biochemically characterized by the accumulation of medium-chain, long-chain hydroxyl, and long-chain fatty acids and derivatives, respectively, in tissues and biological fluids of the affected patients. Clinical manifestations commonly include hypoglycemia, cardiomyopathy, and recurrent rhabdomyolysis. Although the pathogenesis of these diseases is still poorly understood, energy deprivation secondary to blockage of fatty acid degradation seems to play an important role. However, recent evidence indicates that the predominant fatty acids accumulating in these disorders disrupt mitochondrial functions and are involved in their pathophysiology, possibly explaining the lactic acidosis, mitochondrial morphological alterations, and altered mitochondrial biochemical parameters found in tissues and cultured fibroblasts from some affected patients and also in animal models of these diseases. In this review, we will update the present knowledge on disturbances of mitochondrial bioenergetics, calcium homeostasis, uncoupling of oxidative phosphorylation, and mitochondrial permeability transition induction provoked by the major fatty acids accumulating in prevalent FAOD. It is emphasized that further *in vivo* studies carried out in tissues from affected patients and from animal genetic models of these disorders are necessary to confirm the present evidence mostly achieved from *in vitro* experiments.

## Fatty Acid Oxidation Defects

Mitochondrial fatty acid oxidation is critical to provide ATP to mitochondria-enriched tissues with high energy demand, including the heart, skeletal muscle, and liver. Mutations in genes expressing enzymes or transport proteins involved in this catabolic pathway cause the fatty acid oxidation defects (FAOD) that are biochemically characterized by accumulation of specific patterns of fatty acids and acylcarnitine derivatives. Patients typically manifest hypoglycemia, cardiomyopathy, hepatopathy, recurrent rhabdomyolysis, and encephalopathy, whose pathogenesis is still poorly known, although energy deprivation secondary to blockage of fatty acid degradation seems to play an important role.

Deficiencies of medium-chain acyl-CoA dehydrogenase (MCAD), mitochondrial trifunctional protein (MTP), isolated long-chain 3-hydroxyacyl-CoA dehydrogenase (LCHAD), and very long-chain acyl-CoA dehydrogenase (VLCAD) are the most common of these diseases. During acute episodes of metabolic decompensation usually associated with infections or fasting, the affected patients present hypoglycemia, encephalopathy, cardiomyopathy, rhabdomyolysis, and hepatopathy. Interestingly, during these catabolic crises, there is a high release of fatty acids from the adipose tissue and a significant increase of the accumulating fatty acids and derivatives due to the enzymatic defects, therefore suggesting the toxicity of these compounds (Rinaldo et al., 2002; Kompare and Rizzo, 2008; Olpin, 2013; Knottnerus et al., 2018; Anderson et al., 2020).

Medium-chain acyl-CoA dehydrogenase-deficient patients are generally asymptomatic, but 20–40% of them develop severe symptomatology along life during an acute episode of metabolic decompensation commonly triggered by prolonged fasting or infections (Merritt and Chang, 2019). During crises, they present hypoketotic hypoglycemia, vomiting that may progress to seizures and coma as well as elevation of liver enzymes and hepatomegaly, whereas a considerable percentage of them may have a fatal outcome (Grosse et al., 2006; Merritt and Chang, 2019; Anderson et al., 2020). Recurrent rhabdomyolysis is remarkably rare in this disease (Ruitenbeek et al., 1995; Schatz and Ensenauer, 2010), although long-term neurological complications are observed in up to 10–30% of patients (Maier, 2015). Tissue (blood) accumulation of medium-chain fatty acids (MCFA), especially octanoic acid (OA), decanoic acid (DA), and cis-4-decenoic acid (cDA), as well as their corresponding acylcarnitine derivatives hexanoylcarnitine, octanoylcarnitine (OC), decanoylcarnitine (DC), and cis-4-decenoylcarnitine and acylglycines (hexanoylglycine, suberylglycine, and phenylpropionylglycine) in urine is detected especially during episodes of acute metabolic decompensation (Dobrowolski et al., 2017; Anderson et al., 2020).

Mitochondrial trifunctional protein comprises three enzyme activities that catalyze the second, third, and fourth step of mitochondrial fatty acid oxidation cycle, namely, long-chain enoyl-CoA hydratase, LCHAD, and long-chain ketoacyl-CoA thiolase. Two inherited diseases are caused by defects of this complex protein, the isolated LCHAD deficiency and MTP deficiency. Long-chain 3-hydroxyacyl-CoA dehydrogenase deficiency is caused by a common mutation (c.1528 G > C) in the HADHA gene, whereas all other HADHA and HADHB mutations in this complex enzyme lead to MTP deficiency (De Biase et al., 2017).

Untreated MTP and LCHAD deficiencies have high mortality and morbidity. The clinical features are usually manifested during fasting or metabolic stress triggered by common illness and mainly affect the heart and skeletal muscles that are highly dependent on fatty acids for their energy needs (Sykut-Cegielska et al., 2011; De Biase et al., 2017; Lotz-Havla et al., 2018). Individuals with MTP deficiency usually present a severe neonatal early onset form with elevated mortality caused by cardiomyopathy, as well as peripheral neuropathy, whereas patients with LCHAD deficiency commonly have a moderate late-onset phenotype with cardiomyopathy, retinal disease, and peripheral neuropathy during adulthood. Patients affected by these diseases may also present with hepatopathy and episodes of rhabdomyolysis during situations of intense lipolysis (Rocchiccioli et al., 1990; Tyni et al., 1997; Den Boer et al., 2002, 2003; Moczulski et al., 2009; De Biase et al., 2017). Long-term complications include rhabdomyolysis, cardiomyopathy, peripheral neuropathy, and retinopathy (Karall et al., 2015; Lotz-Havla et al., 2018). High levels of the long-chain 3-hydroxy fatty acids (LCHFA), 3-hydroxytetradecanoic acid (3HTA), 3-hydroxypalmitic acid (3HPA), and 3-hydroxytetradecanedioic acid (3HTDA) and their respective carnitine by-products accumulate in the patients, with 3-hydroxypalmitoylcarnitine (C16–OH) and 3-hydroxyoleoylcarnitine (C18:1–OH) being the primary biomarkers characteristically found at high concentrations in their blood (Hagenfeldt et al., 1990; Costa et al., 1998; Jones et al., 2001; Hintz et al., 2002; Olpin, 2005; Sander et al., 2005). High amounts of triglycerides containing long-chain fatty acids (LCFA) have been also shown in LCHAD-deficient patients (McCoin et al., 2016).

Very long-chain acyl-CoA dehydrogenase is the first and rate-limiting intramitochondrial step in the mitochondrial oxidation of LCFA. The deficiency of this enzyme activity causes the most prevalent defect of LCFA β-oxidation. The affected patients commonly manifest hypoglycemia, cardiomyopathy, and recurrent rhabdomyolysis. Hypoglycemia usually occurs during 2–4 years of life, whereas rhabdomyolysis is manifested later, and cardiomyopathy can occur at any age. Patients may present three different clinical phenotypes: (a) a severe early onset manifestation with life-threatening cardiomyopathy associated with arrhythmias, hypotonia, hepatomegaly, and intermittent hypoglycemia, (b) an early childhood presentation with hypoketotic hypoglycemia associated with liver alterations and hepatomegaly, or (c) a late-onset myopathic form with recurrent episodes of rhabdomyolysis and muscle pain usually induced by exercise (Spiekerkoetter, 2010; Diekman et al., 2014; Katz et al., 2017; Leslie et al., 2019; Rovelli et al., 2019). Recurrent rhabdomyolysis is a common and acute complication of this disease and should be treated with hydration and alkalization of the urine to prevent acute renal failure secondary to myoglobinuria. Elevated levels of myristic (Myr) and cis-5-tetradecenoic (Cis-5) acids, as well as their acylcarnitine derivatives tetradecenoyl-L-carnitine (C14:1), tetradecadienyl-L-carnitine (C14:2), tetradecanoyl-L-carnitine (C14), and dodecanoyl-L-carnitine (C12), are commonly found in blood (McHugh et al., 2011).

The diagnosis of MCAD, LCHAD/MTP, and VLCAD deficiencies is mainly performed by the detection of high concentrations of characteristic acylcarnitines in blood. The determination of enzyme activities in lymphocytes and/or fibroblasts and molecular analyses of mutations may be necessary for diagnosis confirmation. Since early diagnosis and prompt treatment are available in MCAD, LCHAD/MTP, and VLCAD deficiencies, these diseases have been included in the expanded newborn screening programs, allowing a much better outcome for the affected patients by significantly reducing morbidity and mortality (Wilcken et al., 2007; Spiekerkoetter et al., 2010; Maguolo et al., 2020).

The current treatments for these diseases include frequent meals and avoidance of catabolic stress situations caused by prolonged fasting or infectious illness. Fatty acid restriction, allied to medium-chain triglycerides (MCT) formulas and essential fatty acids to LCHAD/MTP and VLCAD deficiencies, as well as high oral or intravenous glucose administration to sustain anabolism is also critical in these disorders. The objective is therefore preventing hypoketotic hypoglycemia and metabolite accumulation. L-Carnitine supplementation should be used mainly to correct L-carnitine deficiency, but its beneficial effect to significantly increase the urinary excretion of potentially toxic fatty acids has still to be demonstrated (Spiekerkoetter et al., 2009, 2010). More recently, clinical trials have shown that bezafibrate, an agonist of peroxisome-proliferating activator receptor that increases mitochondrial biogenesis and the gene expression of mitochondrial fatty acid oxidation enzymes, may be useful in VLCAD (Yamada et al., 2018; Shiraishi et al., 2019) and MTP (Suyama et al., 2020) deficiencies. However, the clinical efficacy of bezafibrate is still disputed and needs to be further confirmed (Ørngreen et al., 2014, 2015). Furthermore, replacement of long-chain triglycerides by MCT and replenishment of citric acid cycle (CAC) intermediates by the seven-carbon fatty acid triglyceride (C7) triheptanoin to support ATP production have been recently demonstrated to improve the clinical outcome of these patients (Gillingham et al., 2017; Vockley et al., 2017, 2019).

Treatment is effective to decrease mortality and morbidity for most FAOD, although it does not completely prevent long-term systemic and neurological complications. It is therefore expected that elucidation of the exact underlying mechanisms of pathogenesis of these disorders will potentially help in the development of novel treatments to improve the quality of life of the affected patients. In particular, observations of mitochondrial biochemical and morphological abnormalities in highly mitochondria-enriched tissues of MCAD-, LCHAD/MTP-, and VLCAD-deficient patients, such as the heart, liver, and skeletal muscle, indicate that disturbances of mitochondrial functions are probably involved in their pathophysiology. The present review will mainly focus on recent evidence indicating secondary alterations of important mitochondrial properties caused by major fatty acids accumulating in these disorders.

## Mitochondrial Functions: Bioenergetics and Calcium Homeostasis

The mitochondria are dynamic organelles with essential roles in cellular physiology, particularly in tissues with high energy demand and oxidative metabolism, such as the heart, skeletal muscle, liver, and brain. The mitochondria regulate ATP production, redox status, cytosolic calcium concentrations, and apoptosis-induced cell death. These mitochondrial functions are closely associated with efficient dynamic processes, such as mitochondrial fission and fusion, biogenesis, and mitophagy.

Mitochondrial fatty acid oxidation is the main source of ATP generation in the heart, skeletal muscle, and liver. Acetyl-CoA originated from fatty acid degradation is oxidized in the CAC, providing the electrons to form NADH and FADH_2_, which are transferred through the respiratory chain complexes in the electron transfer system. Protons are pumped into the mitochondrial intermembrane space, generating an electrochemical gradient (mitochondrial membrane potential, ΔΨm). The energy of the electrochemical gradient is used by ATP synthase to support ATP production, and the whole process is called oxidative phosphorylation (OXPHOS; Nicholls and Ferguson, 2013).

Calcium regulates a significant number of critical intracellular events necessary to cell survival, particularly in the heart, skeletal muscle, and liver (Rizzuto et al., 2012). The mitochondria continuously promote calcium influx down the electrochemical gradient through the mitochondrial calcium uniporter (MCU) and efflux by the Na^+^/Ca^2+^ and H^+^/Ca^2+^ exchangers, contributing together with the endoplasmic reticulum to maintain the cytosolic concentrations of this ion that would be adequate for cell functioning (De Stefani et al., 2016). At physiological or pathological situations of calcium overload, the capacity of the mitochondria to retain calcium is particularly important (Williams et al., 2013; Granatiero et al., 2017; Nicholls, 2017).

Mitochondrial permeability transition (mPT) pore opening in the inner mitochondrial membrane occurs when the threshold of mitochondrial calcium retention capacity is exceeded. Mitochondrial permeability transition pore opening is deleterious to cell functioning and survival mainly due to non-selective mitochondrial permeabilization that leads to significant calcium, glutathione, and NADH and NAD(P)H release, inducing mitochondrial swelling, ΔΨm dissipation, disruption of ATP synthesis, and finally apoptotic cell death (Rasola and Bernardi, 2011; Bernardi and von Stockum, 2012; Giorgio et al., 2018).

Mitochondrial calcium homeostasis represents an important regulatory mechanism in muscular tissue physiology, being extremely important for normal cardiomyocyte (Drago et al., 2012; Eisner, 2014; Kohlhaas et al., 2017) and myocyte (Yi et al., 2011; Eisner, 2014) functioning. Dysregulation of intracellular calcium concentration due to mitochondrial dysfunction associated with mPT induction has been related to cardiac diseases (Gordan et al., 2016; Bravo-Sagua et al., 2017) and suggested as a pathophysiologic event leading to rhabdomyolysis (Hamel et al., 2015). Disturbance of mitochondrial calcium homeostasis and mPT pore opening has also been demonstrated to be involved in liver diseases (Ferreira et al., 2003; Brenner et al., 2013; Go et al., 2015).

On the other hand, balanced mitochondrial fusion (elongation) and fission (fragmentation) is essential for normal mitochondrial morphology, distribution, and function, being also necessary in response to the variable physiological demands of the cell (El-Hattab et al., 2018). These processes can restore or remove defective mitochondria and are mainly coordinated by the pro-fusion mitochondrial proteins optic atrophy 1 (OPA1), mitofusin 1 (MFN1), and mitofusin 2 (MFN2), as well as by the cytosolic pro-fission dynamin-related protein 1 (DRP1; Burté et al., 2015). The quantity and the quality of the mitochondria are regulated by the equilibrium between their formation (biogenesis) and removal (mitophagy). Upregulation of biogenesis improves mitochondrial function and has been related to various proteins, such as the peroxisome proliferator-activated receptor gamma coactivator 1-alpha (PGC-1α) and sirtuin 1 (SIRT1), whereas mitophagy is dependent on the PTEN-induced putative kinase 1 (PINK1; Ploumi et al., 2017). PTEN-induced putative kinase 1 is highly expressed in injured mitochondria with collapsed ΔΨm, recruiting and activating mitophagy-related proteins, particularly ubiquitin ligase parkin. It is noteworthy that the defects in mitochondrial dynamics result in the impairment of mitochondrial bioenergetics and calcium homeostasis, potentially leading to cell death (Pernas and Scorrano, 2016; Kowaltowski et al., 2019). Furthermore, failure in mitochondrial dynamics has been related to various pathological processes, including myopathies, cardiomyopathy, hepatopathy, neurodegeneration, diabetes, and cancer (Archer, 2013; Vásquez-Trincado et al., 2016; Bartsakoulia et al., 2018; Mansouri et al., 2018; Ji and Yeo, 2019).

## Fatty Acid Oxidation Defects and Mitochondrial Abnormalities

Table 1 summarizes the biochemical and morphological evidence of mitochondrial abnormalities in patients, genetic knockout mice, and cultured cell models of MCAD, LCHAD/MTP, and VLCAD deficiencies.

**TABLE 1:** Mitochondrial biochemical and morphological abnormalities observed in patients and genetic models of MCAD, LCHAD/MTP and VLCAD deficiencies.

**FAOD**		**Evidence of mitochondrial abnormalities**		**References**
MCAD deficiency	Patients	Fibroblasts	↓ Mitochondrial oxygen consumption ↓ Respiratory chain complexes protein levels	Lim et al., 2018
		Blood	Lactic acidemia ↑ Oxidative stress	Feillet et al., 2003 Derks et al., 2014; Najdekr et al., 2015
		Skeletal muscle	Rhabdomyolysis	Ruitenbeek et al., 1995; Schatz and Ensenauer, 2010
	Genetic model	MCAD knockout 143B osteosarcoma cells	↓ Mitochondrial oxygen consumption ↓ Respiratory chain complexes protein levels	Lim et al., 2018
LCHAD/MTP deficiencies	Patients	Fibroblasts	↓ Mitochondrial oxygen consumption ↓ ATP synthesis ↓ MFN2/DRP1 ratio (increased fission) ↑ ROS production	Lefort et al., 2017 Lefort et al., 2017 Hagenbuchner et al., 2018 Hagenbuchner et al., 2018
		Blood	Lactic acidemia	Tyni et al., 1996; Ventura et al., 1998; Das et al., 2000; Enns et al., 2000
		Skeletal muscle	Rhabdomyolysis Mitochondrial abnormalities and respiratory chain inhibition	Olpin, 2005; Diekman et al., 2014 Tyni et al., 1996; Enns et al., 2000; Das et al., 2000
	Genetic model	Mouse liver	↑ Oxidative stress Mitochondrial swelling and distortion	Ibdah et al., 2005 Ibdah et al., 2001
VLCAD deficiency	Patients	Fibroblasts	↓ Mitochondrial oxygen consumption ↓ ATP synthesis ↑ ROS generation ↑ MFN1 levels (increased fusion)	Seminotti et al., 2019
		Cardiomyocytes	↑ Intracellular calcium concentrations	Knottnerus et al., 2020
		Blood	Lactic acidemia	Ventura et al., 1998
		Skeletal muscle	Rhabdomyolysis	Roe et al., 2002; Engbers et al., 2005; Diekman et al., 2014
	Genetic model	Mouse brown adipose tissue	↑ Resting respiration (uncoupling of OXPHOS)	Exil et al., 2006
		Mouse heart	↓ Citric acid cycle intermediates ↓ Phosphocreatine/ATP ratio ↓ ATP production	Bakermans et al., 2013; Gaston et al., 2020 Tucci et al., 2014 Xiong et al., 2014

*DRP1: dynamin-related protein 1; FAOD: fatty acid oxidation defects; LCHAD: long-chain 3-hydroxyacyl-CoA dehydrogenase; MCAD: medium-chain acyl-CoA dehydrogenase; MTP: mitochondrial trifunctional protein; MFN1: mitofusin 1; MFN2: mitofusin 2; ROS: reactive oxygen species; OXPHOS: oxidative phosphorylation; VLCAD: very long-chain acyl-CoA dehydrogenase.*

Increased lactate formation commonly results from the impairment of mitochondrial bioenergetics, with lactic acidosis being therefore considered a biochemical hallmark of mitochondrial disorders (Kerr, 1991; Zeviani and Di Donato, 2004). However, it should be noted that, apart from being indicative of altered mitochondrial functions, lactic acidemia may be also a consequence of decreased lactate utilization due to liver dysfunction as found in some FAOD, particularly during episodes of metabolic decompensation. Patients with these diseases also present episodes of rhabdomyolysis that signalize severe disturbance of mitochondrial functions (Nance and Mammen, 2015). Oxidative stress has been also associated with mitochondrial dysfunction (Wang et al., 2014; Kudryavtseva et al., 2016; Rose et al., 2018) due to impairment of the mitochondrial electron flow through the respiratory chain, resulting in increased electron loss and subsequent reactive oxygen species (ROS) generation (Lambert and Brand, 2009; Nickel et al., 2014).

Although uncommon, lactic acidosis (Feillet et al., 2003), oxidative stress (Derks et al., 2014; Najdekr et al., 2015), and rhabdomyolysis (Ruitenbeek et al., 1995; Schatz and Ensenauer, 2010) have been observed in some patients with MCAD deficiency, particularly during episodes of metabolic decompensation. Reduced mitochondrial oxygen consumption, decreased respiratory chain complex protein levels, and increased ROS production were also found in fibroblasts from MCAD-deficient patients and in MCAD knockout 143B osteosarcoma cells (Lim et al., 2018), supporting a role for the disruption of bioenergetics and of redox homeostasis in the pathogenesis and progression of this disorder.

On the other hand, abnormal mitochondrial morphology and altered biochemical markers of mitochondrial functions, reflecting an impairment of bioenergetics, were commonly reported in isolated LCHAD and MTP deficiencies. In this scenario, lactic acidemia (Tyni et al., 1996; Ventura et al., 1998; Das et al., 2000; Enns et al., 2000), as well as rhabdomyolysis (Olpin, 2005; Diekman et al., 2014), mitochondrial morphological abnormalities, and inhibition of the respiratory chain electron flow (Tyni et al., 1996; Das et al., 2000; Enns et al., 2000; Hintz et al., 2002), was observed in skeletal muscle and cultured fibroblasts from patients with these diseases. Decreased mitochondrial oxygen consumption and reduced ATP synthesis were also detected in the fibroblasts of MTP-deficient patients (Lefort et al., 2017). Furthermore, an interesting study utilizing LCHAD-deficient fibroblasts demonstrated a dysregulation of the mitochondrial fusion/fission machinery as revealed by a decreased MFN2/DRP1 ratio, leading to the accumulation of fragmented mitochondria due to increased fission (Hagenbuchner et al., 2018). Mitochondrial swelling and distortion (Ibdah et al., 2001), as well as induction of oxidative stress, have been also reported in the liver of LCHAD-deficient mice (Ibdah et al., 2005).

As regards to VLCAD deficiency, lactic acidosis (Ventura et al., 1998) and rhabdomyolysis (Roe et al., 2002; Engbers et al., 2005; Diekman et al., 2014) were found in a considerable number of patients with this disorder. Decreased mitochondrial respiration associated with low ATP levels, as well as increased ROS generation, was verified in the fibroblasts of VLCAD-deficient patients (Seminotti et al., 2019). These fibroblasts revealed elevated mitochondrial mass and fusion, as well as an increased expression of MFN1, indicating alterations of mitochondrial dynamics besides the disruption of the endoplasmic reticulum–mitochondria crosstalk that is involved in cytosolic calcium homeostasis (Seminotti et al., 2019). This is in line with the observations of increased intracellular calcium concentrations that were correlated with fatty acid intermediate accumulation in fibroblasts from VLCAD-deficient patients differentiated into cardiomyocytes (Knottnerus et al., 2020). Furthermore, results obtained in the genetic mice model of VLCAD deficiency showed bioenergetics disruption. Thus, increased resting respiration suggesting uncoupling of OXPHOS was found in brown adipose tissue (Exil et al., 2006), and decreased phosphocreatine/ATP ratio (Tucci et al., 2014), ATP production (Xiong et al., 2014), and CAC intermediate pools (Bakermans et al., 2013; Gaston et al., 2020) were demonstrated in the heart of these animals.

## Disturbance of Mitochondrial Bioenergetics and Calcium Homeostasis Caused by the Major Fatty Acids Accumulating in Fatty Acid Oxidation Defects

The pathophysiology of tissue damage in patients with MCAD, LCHAD/MTP, and VLCAD deficiencies has not yet been well established, although energy deprivation caused by fatty acid oxidation blockage, leading to hypoketotic hypoglycemia and sequestration of CoA and L-carnitine, was presumed to be mainly involved in the pathogenesis of these disorders (Spiekerkoetter and Wood, 2010; Olpin, 2013). More recently, alternative pathogenetic mechanisms have been hypothesized to contribute to their symptomatology, more particularly the toxicity of the accumulating metabolites. This is in accordance with the observations that clinical worsening occurs during stress catabolic situations characterized by intense lipolysis leading to the massive production of these compounds (Gregersen and Olsen, 2010; Olpin, 2013; Tein, 2013; Knottnerus et al., 2018).

In particular, there is mounting evidence of bioenergetics impairment in tissues that primarily consume fatty acids for their energy needs in patients and in the genetic models of MCAD, LCHAD/MTP, and VLCAD deficiencies (Table 1). Tables 2–4 summarize the updated data showing that the major metabolites, particularly MCFA, LCHFA, and LCFA, accumulating in these disorders provoke alterations of mitochondrial functions in the heart, liver and skeletal muscle of rats and also in cultured cell lines, mainly impairing mitochondrial bioenergetics and calcium homeostasis by distinct mechanisms. It is emphasized that, overall, the doses of the accumulating metabolites used in most of these studies were similar to the levels described in the blood of affected patients during metabolic decompensation. However, a significant impairment of mitochondrial functions was also achieved with lower concentrations of these compounds in LCHAD/MTP and VLCAD deficiencies that better approach the levels found in blood during periods of stable disease, therefore suggesting the chronic toxicity of these compounds in these inherited deficiencies.

**TABLE 2:** Major metabolites accumulating in MCAD deficiency disturb mitochondrial bioenergetics and calcium homeostasis in liver and skeletal muscle of rats, as well as in cultured cell lines.

**FAOD**	**Accumulating metabolites**	**Disturbance of mitochondrial bioenergetics and calcium homeostasis**		**References**
MCAD deficiency	OA	Liver supernatants	↓ Complexes I-III, II-III and IV activities ↑ Oxidative stress ↓ ATP/O ratio	Scaini et al., 2012 Scaini et al., 2012 Gallis et al., 2007
		Skeletal muscle supernatants	↓ Complex IV activity ↑ Oxidative stress induction	Scaini et al., 2012
		Hepatocytes	↓ΔΨm	Rial et al., 2018
		Adipocytes	↑ Apoptosis	Yang et al., 2009
	OC	Liver mitochondria	No alterations	Amaral et al., 2016
		Fibroblasts	No alterations	Lefort et al., 2017
	DA	Liver supernatants	↓ Complexes I-III, II-III and IV activities ↑ Oxidative stress	Scaini et al., 2012
		Skeletal muscle supernatants	↓ Complex IV activity ↑ Oxidative stress	Scaini et al., 2012
		Liver mitochondria	↓ Complexes II-III and IV activities ↑ Oxidative stress ↓ ATP-linked and maximal respiration ↑ Resting respiration (uncoupling of OXPHOS) ↓ΔΨm and matrix NAD(P)H concentrations ↓ Calcium retention capacity Induction of mPT pore opening	Amaral et al., 2016
		Hepatocytes	↓ΔΨm	Rial et al., 2018
		Adipocytes	↑ Apoptosis	Yang et al., 2009
	DC	Liver mitochondria	No alterations	Amaral et al., 2016
		Fibroblasts	No alterations	Lefort et al., 2017
	cDA	Liver mitochondria	↓ Complexes II-III and IV activities ↑ Oxidative stress ↓ ATP-linked and maximal respiration ↑ Resting respiration (uncoupling of OXPHOS) ↓ΔΨm and matrix NAD(P)H concentrations ↓ Calcium retention capacity Induction of mPT pore opening	Amaral et al., 2016

*cDA: cis-4-decenoic acid; DA: decanoic acid; DC: decanoylcarnitine; FAOD: fatty acid oxidation defects; MCAD: medium-chain acyl-CoA dehydrogenase; ΔΨm: mitochondrial membrane potential; mPT: mitochondrial permeability transition; OA: octanoic acid; OC: octanoylcarnitine; OXPHOS: oxidative phosphorylation.*

**TABLE 3:** Major metabolites accumulating in LCHAD/MTP deficiencies disturb mitochondrial bioenergetics and calcium homeostasis in heart, liver and skeletal muscle of rats, as well as in cultured cell lines.

**FAOD**	**Accumulating metabolites**	**Disturbance of mitochondrial bioenergetics and calcium homeostasis**		**References**
LCHAD/MTP deficiencies	3HTDA	Heart, liver and skeletal muscle mitochondria	No alterations	Hickmann et al., 2015; Cecatto et al., 2015; Cecatto et al., 2016
	3HTA/3HPA	Heart mitochondria	↓ΔΨm and matrix NAD(P)H concentrations ↑ Swelling ↓ Calcium retention capacity ↓ ATP production - Induction of mPT pore opening	Cecatto et al., 2015
		Cardiomyocytes	↓ ATP-linked and maximal respiration ↑ Resting respiration (uncoupling of OXPHOS)	Cecatto et al., 2018b
		Liver mitochondria	↓ ATP-linked and maximal respiration ↑ Resting respiration (uncoupling of OXPHOS) ↓ΔΨm and matrix NAD(P)H concentrations ↓ Calcium retention capacity ↑ Swelling Induction of mPT pore opening	Hickmann et al., 2015
		Hepatocytes	↓ ATP-linked and maximal respiration ↑ Resting respiration (uncoupling of OXPHOS)	Cecatto et al., 2018b
		Skeletal muscle mitochondria	↓ ATP-linked and maximal respiration ↑ Resting respiration (uncoupling of OXPHOS) ↓ΔΨm and matrix NAD(P)H concentrations ↓ Calcium retention capacity ↓ Mitochondrial membrane fluidity Induction of mPT pore opening	Cecatto et al., 2016
		Skeletal muscle fibers	↓ ATP-linked and maximal respiration	Cecatto et al., 2016

*FAOD: fatty acid oxidation defects; 3-HTDA: 3-hydroxytetradecanedioic acid; 3-HTA: 3-hydroxytetradecanoic acid; 3HPA: 3-hydroxypalmitic acid; LCHAD: long-chain 3-hydroxyacyl-CoA dehydrogenase; ΔΨm: mitochondrial membrane potential; mPT: mitochondrial permeability transition; MTP: mitochondrial trifunctional protein; OXPHOS: oxidative phosphorylation.*

**TABLE 4:** Major metabolites accumulating in VLCAD deficiency disturb mitochondrial bioenergetics and calcium homeostasis in heart, liver and skeletal muscle of rats, as well as in cultured cell lines.

**FAOD**	**Accumulating metabolites**	**Disturbance of mitochondrial bioenergetics and calcium homeostasis**		**References**
VLCAD deficiency	Myr/Cis-5	Heart mitochondria	↓ Complex I activity ↓ ATP-linked and maximal respiration ↑ Resting respiration (uncoupling of OXPHOS) ↓ΔΨm and matrix NAD(P)H concentrations ↓ ATP production ↓ Calcium retention capacity Induction of mPT pore opening	Cecatto et al., 2018a
		Heart fibers	↓ ATP-linked and maximal respiration	Cecatto et al., 2018a
		Cardiomyocytes	↓ ATP-linked and maximal respiration ↑ Resting respiration (uncoupling of OXPHOS) ↓ΔΨm	Cecatto et al., 2018a
		Liver mitochondria	↓ Complex I-III activity ↓ ATP-linked and maximal respiration ↑ Resting respiration (uncoupling of OXPHOS) ↓ΔΨm ↓ ATP production ↑ Swelling ↑ Cytochrome c release ↓ Calcium retention capacity Induction of mPT pore opening	Cecatto et al., 2020 Cecatto et al., 2020 Cecatto et al., 2020; Wieckowski and Wojtczak, 1998; Bodrova et al., 2000 Cecatto et al., 2020 Bodrova et al., 2003; Cecatto et al., 2020
		Hepatocytes	↓ ATP-linked and maximal respiration ↑ Resting respiration (uncoupling of OXPHOS)	Cecatto et al., 2020
		Skeletal muscle mitochondria	↓ Complex I-III and α-KGDH activity ↓ ATP-linked and maximal respiration ↑ Resting respiration (uncoupling of OXPHOS) ↓ΔΨm ↓ ATP production ↓ Calcium retention capacity Induction of mPT pore opening	Cecatto et al., 2019
		Skeletal muscle fibers	↓ ATP-linked and maximal respiration ↑ Resting respiration (uncoupling of OXPHOS)	Cecatto et al., 2019
	C14:1/C16:1	Cardiomyocytes	Induction of apoptosis and necrosis	Hoffmann et al., 2014
	LCAC	Fibroblasts	↓ Resting respiration ↓ΔΨm	Lefort et al., 2017 Nguyen et al., 2017
		Heart mitochondria	↓ ATP-linked respiration ↑ ROS generation ↓ Calcium retention capacity	Liepinsh et al., 2016 Baydoun et al., 1988; De Villiers and Lochner, 1986
		Cardiomyocytes	Disturbance of cell calcium homeostasis	Berezhnov et al., 2008
		Myocytes	Disturbance of cell calcium homeostasis	McCoin et al., 2015b
		Heart sarcoplasmic reticulum	Disturbance of cell calcium homeostasis	Yamada et al., 2000

*Cis-5: cis-5-tetradecenoic acid; FAOD: fatty acid oxidation defects; LCAC: long-chain acylcarnitines; ΔΨm: mitochondrial membrane potential; mPT: mitochondrial permeability transition; Myr: myristic acid; OXPHOS: oxidative phosphorylation; VLCAD: very long-chain acyl-CoA dehydrogenase.*

Thus, OA, DA, and cDA, the MCFA that most commonly accumulate in MCAD deficiency, were shown to provoke mitochondrial dysfunction in the liver and skeletal muscle of rats. OA and DA were shown to inhibit the activities of the respiratory chain complexes I–III and II–III in rat liver, as well as of complex IV in the liver and skeletal muscle, besides inducing oxidative stress in these tissues (Scaini et al., 2012). In addition, DA and cDA severely inhibited ATP-linked (ADP-stimulated) and maximal (CCCP-stimulated) mitochondrial oxygen consumption, increased resting respiration (induced by the ATP synthase inhibitor oligomycin), and inhibited complexes II–III and IV activities, indicating metabolic inhibition and uncoupling of OXPHOS that lead to energy deficiency besides provoking a disruption of redox homeostasis in isolated liver mitochondria (Amaral et al., 2016). It is noteworthy that, in the same study, it was shown that DA and cDA decreased ΔΨm and matrix NAD(P)H content and stimulated the opening of cyclosporin A-sensitive mPT pore. Calcium retention capacity was also decreased by DA and cDA, probably as a result of mPT induction in the liver mitochondria (Amaral et al., 2016). Other studies reported that OA and DA caused ΔΨm dissipation in hepatocytes (Rial et al., 2018) and induced apoptosis in adipocytes (Yang et al., 2009), whereas OA was demonstrated to decrease the ATP/O ratio in perfused liver, indicating OXPHOS impairment (Gallis et al., 2007). In contrast, the carnitine derivatives of the corresponding MCFA tested, OC and DC, caused no changes in the mitochondrial parameters evaluated, implying that medium-chain acylcarnitine derivatives do not disturb these mitochondrial functions. This is consistent with a recent study showing no alterations of OC and DC on mitochondrial respiration in fibroblasts (Lefort et al., 2017).

As regards to LCHFA, it was demonstrated that 3HTA and 3HPA, which mostly accumulate in tissues and biological fluids of patients affected by LCHAD or MTP deficiencies, decrease ΔΨm, matrix NAD(P)H content, calcium retention capacity, and ATP synthesis besides inducing mitochondrial swelling in calcium-loaded liver and heart mitochondria (Cecatto et al., 2015; Hickmann et al., 2015). It was proposed that these effects were due to the induction of cyclosporin A-sensitive mPT pore opening as well as due to uncoupling and to the metabolic inhibition caused by these fatty acids (Cecatto et al., 2015; Hickmann et al., 2015). Similar results were achieved in cultured cell lines from the heart and liver (Cecatto et al., 2018b), as well as in skeletal muscle mitochondria and permeabilized muscle fibers (Cecatto et al., 2016). Altered mitochondrial membrane fluidity caused by the LCHFA was also shown in the skeletal muscle (Cecatto et al., 2016). However, the corresponding dicarboxylic 3HTDA caused no effect on these parameters, implying a selective toxicity of monocarboxylic LCHFA (Cecatto et al., 2015, 2016; Hickmann et al., 2015).

Disruption of mitochondrial functions has also been suggested as a relevant pathomechanism involved in the cardiomyopathy, hepatopathy, and myopathy in VLCAD deficiency since high concentrations of Myr and Cis-5, which mostly accumulate in this disorder, were shown to be toxic to mitochondrial functions by impairing bioenergetics and calcium homeostasis. In particular, previous works performed in isolated liver mitochondria showed that Myr uncouples OXPHOS through the involvement of the dicarboxylate carrier (Wieckowski and Wojtczak, 1998) and of the ANT (Bodrova et al., 2000). Induction of mPT pore opening was also demonstrated to be caused by Myr (Bodrova et al., 2003). Recent studies demonstrated that, apart from Myr, pathological concentrations of Cis-5 disturb mitochondrial bioenergetics and calcium homeostasis in the heart, liver, and skeletal muscle (Cecatto et al., 2018a, 2019, 2020). These results were obtained in different tissue preparations, such as isolated mitochondria, permeabilized cardiomyocytes and hepatocytes, and heart fibers. Myr and Cis-5 also inhibited complex I–III and α-ketoglutarate dehydrogenase activities and altered mitochondrial respiration, behaving as metabolic inhibitors and uncouplers of OXPHOS, leading to ATP depletion besides reducing ΔΨm and matrix NAD(P)H content. These LCFA also induced cyclosporin A-sensitive mPT in the presence of calcium, causing mitochondrial ΔΨm dissipation, reduction of calcium retention capacity, swelling, and cytochrome c release. These observations are in accordance with a previous study showing that monounsaturated LCFA (C14:1 and C16:1) accumulating in VLCAD deficiency decrease ΔΨm and induce apoptosis and necrosis in cardiomyocytes (Hoffmann et al., 2014). On the other hand, the toxicity of long-chain acylcarnitines (LCAC) was reported to be involved in long-chain FAOD pathogenesis (McCoin et al., 2015a, 2019). In particular, it was demonstrated that LCAC disturb cell calcium homeostasis in myocytes (McCoin et al., 2015b), cardiomyocytes (Berezhnov et al., 2008), and heart sarcoplasmic reticulum (Yamada et al., 2000) besides decreasing resting respiration (Lefort et al., 2017) and provoking ΔΨm dissipation (Nguyen et al., 2017) in fibroblasts. LCAC were also shown to reduce calcium retention capacity (De Villiers and Lochner, 1986; Baydoun et al., 1988), inhibit ATP-linked respiration, and generate ROS in the heart mitochondria (Liepinsh et al., 2016).

**FIGURE 1:**
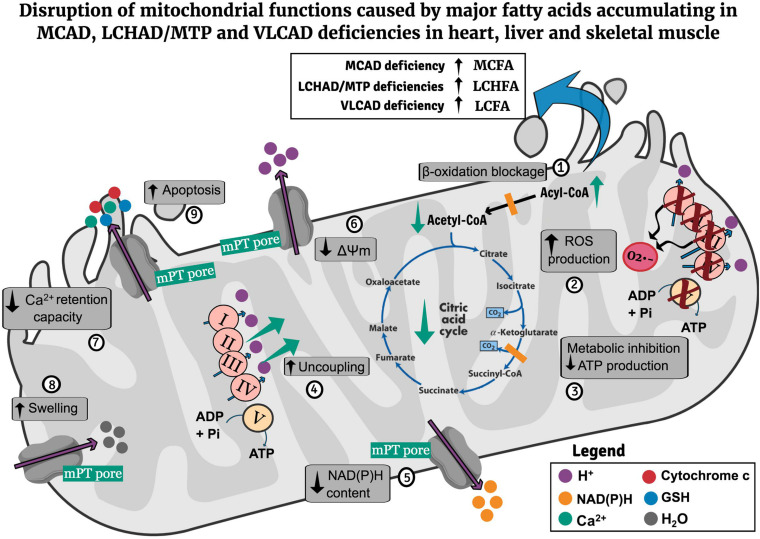
Potential pathomechanisms of mitochondrial dysfunction in the heart, liver, and skeletal muscle in MCAD, LCHAD/MTP, and VLCAD deficiencies. Mitochondrial β-oxidation blockage leads to the matrix accumulation of MCFA (MCAD deficiency), LCHFA (LCHAD/MTP deficiencies), and LCFA (VLCAD deficiency) as well as CoA depletion (1). The accumulating fatty acids inhibit respiratory chain complexes activities, leading to ROS generation (2), cause metabolic inhibition due to decrease of respiratory chain and citric acid cycle activities compromising ATP synthesis (3), uncouple oxidative phosphorylation (4), activate the mPT pore opening, provoking a decrease of mitochondrial NAD(P)H content (5), ΔΨm (6), and mitochondrial Ca^2+^ retention capacity (7), and induce mitochondrial swelling (8). Finally, mPT induction also promotes cytochrome c release, possibly contributing to apoptosis induction (9). CoA, coenzyme A; LCFA, long-chain fatty acids; LCHAD, long-chain hydroxyacyl-CoA dehydrogenase; LCHFA, long-chain 3-hydroxy fatty acids; MCAD, medium-chain acyl-CoA dehydrogenase; MCFA, medium-chain fatty acids; ΔΨm, mitochondrial membrane potential; mPT, mitochondrial permeability transition; MTP, mitochondrial trifunctional protein; ROS, reactive oxygen species; VLCAD, very long-chain acyl-CoA dehydrogenase.

Figure 1 illustrates the main mechanisms of mitochondrial dysfunction caused by major fatty acids that accumulate in MCAD, LCHAD/MTP, and VLCAD deficiencies.

## Concluding Remarks

Mounting evidence of altered mitochondrial morphology, functions, and dynamics has been recently described in various tissues of patients with MCAD, LCHAD/MTP, and VLCAD deficiencies and in the genetic models of these diseases, suggesting that impairment of mitochondrial homeostasis may play a relevant role in the pathogenesis of these disorders. In particular, the major accumulating MCFA, LCHFA, and LCFA were demonstrated to severely disturb mitochondrial bioenergetics and calcium homeostasis *in vitro* in highly oxidative mitochondria-enriched tissues, such as the heart, liver, and skeletal muscle. These fatty acids behaved as metabolic inhibitors, uncouplers of OXPHOS, and inductors of mPT pore opening. It is therefore presumed that these pathomechanisms probably contribute to the mitochondrial alterations observed in patients and animal models with these pathological conditions. Interestingly, severe cardiomyopathy, hepatopathy, and skeletal muscle alterations are mainly manifested during catabolic stress situations in which the concentrations of the characteristic fatty acids dramatically increase in tissues and biological fluids, therefore supporting an acute toxicity for these endogenous compounds. However, the present evidence of bioenergetics disruption caused by the accumulating metabolites must be interpreted with caution since most available data were achieved by *in vitro* assays. Further studies performed preferentially *in vivo* in animal models and in patients affected by FAOD are therefore necessary to further clarify the underlying mechanisms of tissue damage in these disorders. Finally, it is expected that, besides restricting fat dietary intake and avoiding fasting, drugs that stimulate mitochondrial function such as bezafibrate and the anaplerotic compound triheptanoin may hopefully improve the clinical outcome of the affected patients (Gillingham et al., 2017; Vockley et al., 2017, 2019; Yamada et al., 2018; Shiraishi et al., 2019; Suyama et al., 2020).

## Author Contributions

Both authors planned and wrote the review article.

## Conflict of Interest

The authors declare that the research was conducted in the absence of any commercial or financial relationships that could be construed as a potential conflict of interest.

## Abbreviations

CAC: citric acid cycle
cDA: cis-4-decenoic acid
Cis-5: cis-5-tetradecenoic acid
DA: decanoic acid
DRP1: dynamin-related protein 1
FAOD: fatty acid oxidation disorders
3HTA: 3-hydroxytetradecanoic acid
3HTDA: 3-hydroxytetradecanedioic acid
3HPA: 3-hydroxypalmitic acid
MCAD: medium-chain acyl-CoA dehydrogenase
MCFA: medium-chain fatty acids
MCT: medium-chain triglycerides
LCAC: long-chain acylcarnitines
LCFA: long-chain fatty acids
LCHAD: long-chain 3-hydroxyacyl-CoA dehydrogenase
LCHFA: long-chain 3-hydroxy fatty acids
ΔΨ m: mitochondrial membrane potential
MCU: mitochondrial calcium uniporter
MFN1: mitofusin 1
MFN2: mitofusin 2
MTP: mitochondrial trifunctional protein
mPT: mitochondrial permeability transition
Myr: myristic acid
OA: octanoic acid
OPA1: optic atrophy 1
OXPHOS: oxidative phosphorylation
PGC-1 α: peroxisome proliferator-activated receptor gamma coactivator 1-alpha
PINK1: PTEN-induced putative kinase 1
ROS: reactive oxygen species
SIRT1: sirtuin 1
VLCAD: very long-chain acyl-CoA dehydrogenase.
